# Age-Dependent Differences in Frequent Mental Distress (FMD) of US Older Adults Living in Multigenerational Families versus Living Alone

**DOI:** 10.3390/ijerph20043747

**Published:** 2023-02-20

**Authors:** Debasree Das Gupta, David W. S. Wong

**Affiliations:** 1 Department of Kinesiology and Health Science, Emma Eccles Jones College of Education and Human Services, Utah State University, Logan, UT 84322, USA; 2 Department of Geography and Geoinformation Science, George Mason University, Fairfax, VA 22030, USA

**Keywords:** older adults, multigenerational, living alone, mental health, frequent mental distress (FMD)

## Abstract

Frequent mental distress (FMD) is prevalent among older Americans, but less is known about disparities in FMD of older adults living in multigenerational families versus living alone. We pooled cross-sectional data (unweighted, n = 126,144) from the Behavioral Risk Factor Surveillance System (BRFSS) between 2016 and 2020 and compared FMD (≥14 poor mental health days in the past 30 days = 1; 0 otherwise) of older adults (≥65 years) living in multigenerational families versus living alone in 36 states. After controlling for covariates, findings indicate 23% lower odds of FMD among older adults living in multigenerational households compared to counterparts living alone (adjusted odds ratio (AOR): 0.77; 95% confidence interval (CI): 0.60, 0.99). Findings also show that the reduction in the odds of FMD with each 5 year increase in age was larger among older adults living in multigenerational families by 18% (AOR: 0.56; 95% CI: 0.46, 0.70) compared to older adults living alone (AOR: 0.74; 95% CI: 0.71, 0.77), and this difference was significant at the 5% significance level. Multigenerational living may have a protective association with FMD among older adults. Further research is needed to identify multigenerational family and non-kin factors that translate into mental health advantages for older adults.

## 1. Introduction

Poor mental health is widely prevalent among older adults and is linked to high rates of morbidity and mortality in this group [1,2,3,4,5,6]. Although empirical estimates vary, approximately a quarter to one-third of older Americans report some type of poor mental health condition, which is often closely associated with psychosocial factors, such as social support, loneliness, and isolation [1,7,8,9,10]. With the progressive aging of the US population, improving mental health among older adults is a critical public health priority [2,11,12].

Poor mental health comprises an array of behavioral conditions that include, for instance, anxiety, depression, stress, and/or distress [13]. Among these mental health conditions, frequent mental distress (FMD) is one specific measure that is tracked locally and nationally as part of the Centers for Disease Control and Prevention’s (CDC) surveillance of health-related quality of life (HRQOL) of US adults [14,15]. FMD—defined as “14 or more days of self-reported poor mental health in the past month”—strongly correlates with medical markers of depression and represents the burden of mental illness in population subgroups [14,15,16]. Among older adults, FMD is more prevalent than clinically diagnosed mental disorders, such as anxiety and depression, which largely remain under-diagnosed and under- or unreported in this group [1,2]. 

Thus, estimating FMD among older adults may be a particularly useful approach to capture the larger proportion of those 65 years and older suffering from mental health disorders. However, among a diverse swath of studies examining the prevalence and risk factors of FMD among population subgroups [4,6,7,13,17,18,19,20,21,22,23,24,25,26], only a small fraction focuses on the FMD of older adults [6,7,22,25]. In this latter group, no previous study has examined the prevalence of FMD or age-associated differences of this condition among older adults living in multigenerational family households. This line of inquiry assumes significance for more than one reason. While the coresidence of older adults with adult children and/or grandchildren used to be the historical norm, a progressive decline in multigenerational living was set off and persisted after the first demographic transition of the US [27,28]. However, recent studies reveal an increasing trajectory in multigenerational living with one in five US older adults living in multigenerational family households [27,29,30,31]. The rising share of multigenerational living of the US older adult population places the spotlight on this demographic group [27,29]. 

The broader literature conceptualizing multigenerational living is currently divided between those that forward benefits versus those that highlight the disadvantages for the mental health of older adults. On the one hand, prior frameworks have emphasized and reported on the salience of multigenerational living for human development and mental well-being over the life span and in healthy aging [32,33,34,35]. Along the same line, age segregation is identified as a factor that limits and hinders mental health in older ages [36,37]. Collectively, these findings suggest that multigenerational living may have the potential to benefit mental health, particularly in older ages. However, on the other hand, challenges, including caregiving stress, intergenerational conflicts and strains, and detriments to the mental health of older adults from multigenerational living, are also highlighted in the literature [28,38,39,40,41,42,43,44,45,46]. Irrespective of this conceptual divide, the empirical evidence on the prevalence of FMD among older adults living in multigenerational families is nonexistent. 

The literature also indicates that the subjective mental health of older adults may be different at different ages. Previous evidence identified resiliency and better mental health in later life compared to younger ages and underscored that the adaptive benefits from better perceived mental health in older ages have a role in healthy aging [2,47,48,49]. Indeed, perceived mental health may have a greater role to play as older adults adjust to and negotiate many later-life changes, including mounting physical health deficits and functional limitations, as well as shrinking occupational roles and geographic spheres of activities [6,50,51]. A prior investigation [6] examining FMD among older adults highlighted age-associated disparities and reported decreased odds of FMD with increasing age. However, the understanding of whether the prevalence and patterns of FMD among older adults in multigenerational families may (or may not) also exhibit advantageous age-associated differences is not known. Lastly, on the other end of the spectrum, the status of living alone has been associated with social isolation, as well as with various adverse health conditions, including poor mental health, in later life [1,11,36,37,51]. 

Therefore, our objective was to evaluate whether multigenerational living has a protective (or a risk) association with FMD among older adults. In so doing, we first estimated whether the likelihood of FMD was lower among older adults living in multigenerational families compared to older adults living alone. Additionally, we also compared and contrasted the age-associated disparities in FMD among older adults living in multigenerational family households against older adults living alone. 

## 2. Materials and Methods

Data: We used data from BRFSS, which is a leading source of nationally representative health data in the US [52,53]. The CDC, together with state health departments, has conducted this annual cross-sectional survey since 1984 in all 50 states, the District of Columbia, Guam, and Puerto Rico [52]. Trained interviewers administer the BRFSS survey via telephone to participants randomly selected from noninstitutionalized adults 18 years and older. A standardized questionnaire is used to track chronic conditions and health risk behaviors, access to and use of health services, and socioeconomics and demographics of noninstitutionalized adults. The BRFSS employs a probability-based multistage cluster sampling methodology to generate representative samples in all 50 US states and regions. Additional details on this complex survey design methodology are available from the CDC [54]. Each year, approximately 400,000 adults participate in the BRFSS, and between 2016 and 2020, the mean response rate was 47.8%. The institutional review board (IRB) at the CDC has approved the BRFSS for research [55]. The data made available to the public are not individually identifiable, and a secondary analysis of this data does not constitute human subject research.

Study sample: The study sample included BRFSS landline participants 65 years and older who were living alone or were living in multigenerational families (unweighted count, n = 172,193; weighted count, N = 36,773,297) in 36 states across the US. The respondents participating via landline were asked the question: “*Excluding adults living away from home, such as students away at college, how many members of your household, including yourself, are 18 years of age or older?*” A second BRFSS question asked participants: “*How many children less than 18 years of age live in your household?*” as part of the Random Child Selection module that was administered in 36 states between 2016 and 2020. We identified all landline participants 65 years and older who responded “adults = 1” and “children = 0” to these two questions as older adults “living alone” (93.9% of the total study sample, n = 167,272; N = 34,546,148) in our analysis. The status of “living alone” has been used in prior studies, including those that use BRFSS data, as an objective indicator to capture the risk of social isolation, lower social support, and poor mental health consequences [50,51,56,57,58,59,60,61,62,63].

Next, to identify multigenerational family households, we applied a framework employed in prior studies [30,43,64,65,66]. Accordingly, we designated “multigenerational” as grandparent–grandchild family structures with or without the middle generation parents present in the household. If a participant reported one or more children (≤17 years) living in the household, the BRFSS asked the respondents: “*How are you related to the child?*”, and “grandparent” was one of the response options. All BRFSS landline respondents 65 years and older who indicated “grandparent” to this question constituted older adults living in multigenerational family households (6.1% of the total study sample, n = 4921; N = 2,227,149) in our study. In prior studies, the BRFSS “relationship” question has been used to identify grandparent–grandchild multigenerational family structures and to investigate older adult health [62,67,68,69].

Of the 172,193 older adult respondents living alone or living in multigenerational families, 73.3% (n = 126,144; N = 26,446,545) had no missing information on the mental health outcome variable and the covariates and comprised the analytic sample in our study. The composition of this analytic sample was (1) multigenerational, 6.5% (n = 3898; N = 1,722,633), and (2) living alone, 93.5% (n = 122,246; N = 24,723,912). This composition was comparable to the proportion of older adults living alone versus living in multigenerational families among excluded participants.

Outcome and explanatory variables: The outcome variable in our analysis was FMD, which was constructed using the “poor mental health days” question included under the BRFSS 4-item Healthy Days Core Module (CDC HRQOL-4) developed by the CDC [70]. Since 1993, the BRFSS has administered this module to ask respondents questions representing the four domains of overall, physical, mental, and social health [70]. The CDC HRQOL-4 questions are established valid measures, with multiple studies attesting to their strong psychometric properties and high test–retest reliability [71,72,73,74,75,76]. The longstanding validity of these measures is also documented in older adult populations, and, in congruence, these questions have been incorporated in the Medicare Health Outcomes Survey since 1995 [77,78,79,80]. 

The “poor mental health days” question asks respondents: “Now thinking about your mental health, which includes stress, depression, and problems with emotions, for how many days during the past 30 days was your mental health not good?” The reference time period of “past 30 days” was included to capture the emerging chronic symptoms impacting HRQOL, and a 14 day cut-point was employed to dichotomize this numeric measure (range: 0 to 30 days) to represent FMD [70,75,81,82]. The 14 day threshold closely mirrors the duration used in clinical research and practice to indicate and diagnose depressive and anxiety disorders [13,71,75,81,82,83,84]. Previous studies on poor mental health have adopted this binary conceptualization as a single-unit measure to capture frequent and persistent deficits in mental HRQOL across various demographics, and applications of FMD are also found in state- and county-level population health surveillance studies [4,6,7,13,17,18,19,20,21,22,23,25,26,85,86,87,88,89]. We adopted this 14 day dichotomous metric to construct the outcome variable representing FMD (≥14 poor mental health days = 1; 0 otherwise) in our analysis.

The status of living in multigenerational families (=1) versus living alone (=0) served as the primary explanatory variable in our study. We also adopted an alternative coding scheme motivated upon findings reported in prior studies [62,67,68,69,90,91]. Previous results have highlighted the unique health vulnerabilities of single or solo grandparents living with their grandchild only [62,67,68,69,90,91]. Thus, we created a secondary explanatory variable consisting of the three categories of older adults living alone (=0), living with their grandchildren only (=1), and living with their grandchildren, spouse, and/or other adults (=2). We used responses to the following BRFSS questions: (1) number of adults 18 years or older in the household = 1, and children less than 18 years of age in the household = 0, coded with a (0) = living alone; (2) number of adults 18 years or older in the household = 1, children less than 18 years of age in the household ≠ 0, and the relationship to child = “grandparent” coded with a (1) = living with grandchild only; and (3) number of adults 18 years or older in the household > 1, children less than 18 years of age in the household ≠ 0, and the relationship to child = “grandparent” coded with a (2) = living with grandchild, spouse, and/or other adults. The BRFSS questions we used to construct this outcome and the explanatory variables are indicated in Table 1 below.

Covariates: Demographics (age, sex, marital status, race, and ethnicity) and socioeconomics (education and income) are significant correlates of individual health including perceived mental and physical health [92]. To adjust for demographic and socioeconomic variations in FMD, we included age (5 year age categories), sex (male or female), marital status (married, unmarried couple, versus divorce, widowed, separated, and never married), race/ethnicity (White non-Hispanic (NH), Black NH, other NH, and Hispanic), education (<high school, high school, attended college or technical school, and ≥college or technical school), and household income (<$15,000, $15,000–24,999, $25,000–34,999, $35,000–49,999, and ≥$50,000) as controls in our analysis.

In addition, chronic conditions, as well as functional, cognitive, and sensory limitations, are reported as significant independent predictors of self-reported mental health status [92]. We combined binary (0 = no/1 = yes) responses to all of the BRFSS questions on lifetime diagnosis of chronic conditions that included depressive disorders; diabetes; cardiovascular diseases: coronary heart disease, myocardial infarction, or stoke; arthritis; chronic kidney disease; asthma; chronic obstructive pulmonary disease; and skin or other cancers to obtain an aggregate number of the chronic health conditions (range: 0–7) of the participants. Based on the US Census’s [93] disability module, the BRFSS includes a six-item questionnaire to ask about functional, cognitive, and sensory limitations. These six questions ask respondents about (1) deafness or having serious difficulty hearing; (2) blindness or having serious difficulty seeing; (3) having serious difficulty concentrating, remembering, or making decisions; (4) having serious difficulty walking or climbing stairs; (5) having difficulty bathing or dressing; and (6) having difficulty doing errands alone, such as visiting a doctor’s office or shopping. We aggregated the binary (no = 0/yes = 1) responses to these six questions in the BRFSS to derive an aggregate number (range: 0–6) representing the “number” of the physical limitations of the participants. Lastly, we included a year dummy variable (2016–2019 = 0 versus 2020 = 1) to adjust for any potential differences in mental health perceptions during the pandemic year.

Statistical Analysis: Based on descriptive statistics (percentages and standard errors), we examined variations in the FMD reported by older adults. Consistent with the methodology used in prior studies, we present the self-reported prevalence of FMD, which is the weighted percentage of respondents indicating FMD [1,4,6]. To control for the influence of confounding variables, we conducted regression analysis in which we considered all of the covariates mentioned above. We estimated the logistic regression specification (Equations (1) and (2) below) to model the binary outcome variable—FMD—employing Stata (version 15, Statacorp LLC, College Station, TX, USA) and conducted all statistical tests using the appropriate methodology for survey data analysis [94]. The findings presented in Section 3 (Results) are the weighted estimates with the statistical significance set at *p* ≤ 0.05 (unless otherwise specified).

(1) $$log\left(\frac{\Pi }{1-\Pi }\right)=\beta_{0}+\beta_{1}X_{1}+\ldots +\beta_{k}X_{k}\;$$

which states that the (natural) logarithm of the odds of an event, referred to as the log odds, is a linear function of the *X* variables, such that:

(2) θ=Π1−Π|X=X1Π1−Π|X=X2

where θ is the odds ratio between the odds for two sets of predictors; *X*_(1)_ and *X*_(2)_, $\left(\frac{\Pi }{1-\Pi }\right)$ are the odds of an event such that Π is the probability of the event, and *X*_1_, …, *X_k_* is the set of predictor variables.

## 3. Results

Descriptive results: Table 2 shows the descriptive characteristics of the two groups of older adults—those living alone versus those living in multigenerational families. Compared to older adults living alone, a higher proportion of older adults in multigenerational families were males (34% versus 31%), younger in age (65–69: 38% versus 19%), and belonged to minority racial ethnic groups (37% versus 19%). Older adults in multigenerational families had lower levels of education (<high school: 19% versus 15%) and lower levels of physical disability (≥1 disability: 47% versus 50%) but comparable levels of chronic health conditions (16% no chronic conditions) when compared to counterparts living alone. 

In Table 3, we provide the self-reported prevalence (weighted %) of FMD among older adults living alone versus living in multigenerational families. The results in Table 3 indicate that the FMD reported by the two groups of older adults were comparable—approximately 9% in each group reported FMD. However, a comparison across 5 year age categories indicated that the FMD reported by older adults living in multigenerational families was lower than those living alone for all age groups, except the 70–74 year old group. This age-associated difference between the FMD of older adults living alone versus living in multigenerational families was statistically significant for the 75 years and older age groups (*p* < 0.05).

Multivariable regression results: In Table 4, we provide the unadjusted (Model 1) and adjusted (Model 2) odds of FMD among older adults. The adjusted model included all covariates presented earlier in Section 2 (Materials and Methods). The findings in Table 4—Model 2 indicate that the odds of FMD reported by older adults living in multigenerational families was lower than that reported by counterparts living alone (adjusted odds ratio (AOR): 0.81; 95% confidence interval (CI): 0.65, 1.02). However, this difference achieved statistical significance only at the 10% significance level.

To further assess the difference in FMD reported by older adults living alone versus living in multigenerational families, we utilized the secondary explanatory variable created using the alternative coding scheme presented in Section 2 (Materials and Methods). This variable consisted of the three categories of older adults living alone (=0), living with their grandchildren only (=1), and living with their grandchildren, spouse, and/or other adults (=2). We refer to this last category as “non-grandchild only multigenerational families” henceforth. The results in Table 4 indicate the unadjusted (Model 3) and adjusted (Model 4) odds of FMD among older adults for these three categories. The unadjusted models (Model 3) shows that older adults living with their grandchild only were more likely to experience FMD compared to older adults living alone. However, this difference was no longer significant after controlling for covariates in the adjusted model (Model 4). Table 4—Model 4 also shows that the odds of FMD among older adults in non-grandchild only multigenerational families was lower by approximately 23% (AOR: 0.77; 95% CI: 0.60, 0.99; *p* < 0.05) when compared to older adults living alone. 

Additionally, the results in both adjusted models (Models 2 and 4) in Table 4 indicate statistically significantly lower odds of FMD with each 5 year increase in age (AOR: 0.73; 95% CI: 0.70, 0.76). To further examine this age-associated difference, we conducted stratified regression analyses and compared the odds of FMD with each 5 year increase in age among older adults in non-grandchild only multigenerational families versus those living alone. The results are presented in Table 5, with Model 1 showing the adjusted odds of FMD among older adults living alone and Model 2 estimating the adjusted odds of FMD among those 65 years and older in non-grandchild only multigenerational families. With each 5 year increase in age, the odds of FMD decreased by 26% (AOR: 0.74; 95% CI: 0.71, 0.77) among older adults living alone (Table 5—Model 1). In contrast, this statistic among older adults in non-grandchild only multigenerational families was a 44% reduction (AOR: 0.56; 95% CI: 0.46, 0.70) in the self-reported odds of FMD with each 5 year increase in age (Table 5—Model 2). 

To evaluate the equality of the coefficients for the 5 year age group variable from the stratified regressions for the two older adult groups—those living alone versus those living in non-grandchild only multigenerational families—we applied the methodology of “seemingly unrelated estimation” for the testing of the equality of the coefficients using the generalized Hausman specification test (Mize, Doan, & Long, 2019; Stata, n.d.). The results of the Hausman test indicated that the coefficients for age (5 year age category) for the two older adult groups were statistically significantly different (*p* < 0.05).

## 4. Discussion

With the progressive decline in physical health and the increase in chronic conditions, perceived mental health has gained salience for the healthy aging of older adults [2,6,47,48,49,50]. Frequent mental distress (FMD) is one of the leading health indicators that represents the burden of poor mental health in population groups [5,14,15,16]. A growing body of work has investigated FMD across various demographics, but studies analyzing FMD among older adults are sparse, and no prior work has examined FMD among older adults living in multigenerational family households. The prevalence of multigenerational living and older grandparent–grandchild coresidence in the US has been rising over recent decades, especially during the COVID-19 pandemic years [27,29,31,95,96]. Prior research conceptualizes the multigenerational living of older adults, on the one hand, to be advantageous and, on the other, to have an adverse influence on later-life mental health and, therefore, is inconclusive [32,33,34,35,38,39,40,41,42,43,44,45,46]. 

We evaluated FMD among older adults living alone versus living in multigenerational families in 36 US states using a representative sample from the BRFSS pooled between 2016 and 2020. In this analysis, the specific research question we aimed to address was, “does multigenerational living have a protective (or a risk) association with FMD among older adults?” In relation, we report two main findings. The results indicate statistically significant (*p* < 0.05) lower likelihood of FMD among older adults living with grandchild, spouse, and/or other adults versus those living alone. The findings also show age-associated differences with the likelihood of FMD decreasing with each 5 year increase in the age of older adults. However, this age-associated decrease in FMD was larger among older adults living with grandchild, spouse, and/or other adults compared to older adults living alone. Together, these results may be indicative of a protective association between multigenerational living and FMD among older adults.

Multiple prior studies forward evidence on the adverse mental health consequences among older adults living alone and, therefore, at an elevated risk of loneliness, social isolation, and feelings of depression [1,11,36,37,51,97]. In conjunction, we extend the prior findings by showcasing the likely benefit of multigenerational living, in contrast to living alone, for FMD among older adults. Our results using FMD are meaningful, as this indicator captures the burden of poor mental health among older adults more accurately than clinical diagnosis of depression [1,2,98].

The findings from our analysis also have some overlap with prior evidence on FMD among older Americans, although no prior study in this area has examined FMD among those 65 years and older living in multigenerational families. Segev, Arif, and Rohrer [6] and Strine, Hootman, Okoro, et al., [4] analyzed BRFSS data and report significantly lower odds of FMD with each 5 year increase in the age of adults 45 years and older. Our results add to this evidence base by revealing that the age-associated reduction in FMD was larger among older adults living with a grandchild, spouse, and/or other adults when compared to counterparts living alone. The literature identifies greater resiliency, lower depressive distress, and lower negative emotions, often despite life challenges and adversities in old age [1,2,47,48,99,100,101,102]. Studies have also shown positive associations between resilience, life satisfaction, and the self-reported mental health of older adults [102,103]. It is plausible that multigenerational living plays a role in the furtherance of later-life resiliency and the mental HRQOL life of older adults. 

The limitations of our study need specific acknowledgement. Given that the BRFSS is a cross-sectional dataset, our analysis could only show an association between the outcome FMD and the explanatory variable—multigenerational living versus status of living alone of older Americans—at one point in time. However, determining the mental health of older adults over time would be critical to establish the role of multigenerational living over the long run using longitudinal data. In addition, based on the BRFSS data, we could not identify features of multigenerational living, for example, family resources and support, which may be particularly beneficial for the mental health and resiliency of older adults. Thus, additional research is needed to parse out the protective features of multigenerational living that translate into a mental health advantage among older adults. Lastly, our analysis constitutes findings on older adults that are generalizable across a subset (approximately two-thirds) of the states in the US. Notwithstanding these limitations, a primary contribution of our study has been to forward evidence on the subjective mental health, specifically frequent mental distress (FMD), of older adults living in multigenerational families using a national dataset.

## 5. Conclusions

The research suggests that better perceived mental health at older ages has a role in healthy aging. An important question is whether this later-life advantage is a characteristic that represents the subjective mental health of older adults living in multigenerational family households. Addressing this question becomes paramount as the share of older adults living in multigenerational family households continues to rise in the US, particularly since the pandemic years. Using a national dataset, we showed a lower likelihood of frequent mental distress among older adults living in multigenerational families compared to older adults living alone in 36 US states. Once formed, multigenerational families often tend to live together over time and, therefore, have the potential to facilitate aging in place, as well as lower the need for the institutional care of older adults. Future studies are needed to identify the specific features of multigenerational living that improve or limit mental health among older adults across life stages and over time.

## Figures and Tables

**Table 1:** Details on the outcome and explanatory variables, and the BRFSS questions used to construct these variables.

Variable Name	Variable Description	Question(s) in BRFSS Survey
Outcome Frequent mental distress (FMD)	FMD: ≥14 poor mental health days = 1; 0 otherwise	Now thinking about your mental health, which includes stress, depression, and problems with emotions, for how many days during the past 30 days was your mental health not good?
Explanatory		
Primary: multi-generational (=1) living versus living alone (=0)	Multigenerational: (relationship to child = “grandparent”) = 1	How are you related to the child? Excluding adults living away from home, such as students away at college, how many members of your household, including yourself, are 18 years of age or older? How many children less than 18 years of age live in your household?
	Living alone: (adults = 1 and children = 0) = 0	
Secondary: living alone (=0) versus living with grandchildren only (=1) versus living with grandchildren, spouse, and/or other adults (=2)	Living alone: (adults = 1 and children = 0) = 0	
	Living with grandchildren only (adults = 1 and children ≠ 0 and relationship to child = “grandparent”) = 1	
	Living with grandchildren, spouse, and/or other adults (adults > 1 and children ≠ 0 and relationship to child = “grandparent”) = 2	

**Table 2:** Descriptive characteristics of older adults living alone versus living in multigenerational families, BRFSS 2016–2020.

	Living Alone		Living in Multigenerational Families	
	n = 122,246; N = 24,723,912		n = 3898; N = 1,722,633	
	%	[95% CI]	%	[95% CI]
**Sex**				
Male	30.7	[30.0, 31.3]	33.7	[30.2, 37.2]
Female	69.3	[68.7, 70.0]	66.4	[62.8, 69.7]
**Age groups**				
65–69	19.4	[18.8, 19.9]	38.3	[35.0, 41.8]
70–74	21.8	[21.2, 22.3]	29.4	[26.4, 32.7]
75–79	21.0	[20.5, 21.6]	19.5	[16.0, 23.5]
80 and above	37.9	[37.2, 38.6]	12.8	[10.3, 15.8]
**Marital Status**				
Married/unmarried couple	6.2	[5.9, 6.5]	54.1	[50.3, 57.8]
Divorced/widowed/separated/never married	93.8	[93.5, 94.1]	45.9	[42.2, 49.7]
**Race/ethnicity**				
Non-Hispanic (NH) White	81.2	[80.5, 81.8]	62.8	[58.8, 66.7]
NH Black	9.6	[9.1, 10.0]	17.6	[14.6, 21.1]
NH other	3.8	[3.3, 4.2]	5.8	[4.5, 7.6]
Hispanic	5.5	[5.1, 5.9]	13.8	[10.6, 17.6]
**Education**				
<High school	14.9	[14.4, 15.5]	19.4	[16.0, 23.2]
High school	33.9	[33.2, 34.5]	30.4	[27.3, 33.8]
Attended college or technical school	31.0	[30.3, 31.6]	34.2	[30.7, 37.9]
≥College or technical school	20.2	[19.8, 20.7]	16.1	[14.1, 18.2]
**Income**				
<$15,000	17.4	[16.8, 18.0]	11.9	[8.9, 15.8]
$15,000–24,999	30.6	[30.0, 31.3]	23.4	[20.2, 26.8]
$25,000–34,999	16.8	[16.3, 17.3]	15.4	[12.8, 18.3]
$35,000–49,999	15.4	[14.8, 15.9]	15.8	[13.7, 18.1]
≥$50,000	19.9	[19.4, 20.4]	33.6	[30.5, 36.9]
**Chronic conditions**				
No	16.8	[16.3, 17.4]	16.2	[13.7, 19.0]
Yes	83.2	[82.6, 83.8]	83.8	[81.0, 86.3]
**Physical limitations**				
No	50.6	[49.9, 51.2]	53.1	[49.4, 56.8]
Yes	49.5	[48.8, 50.1]	46.9	[43.2, 50.6]

n, Unweighted observation count; N, weighted observation count; CI, confidence interval; %, weighted %.

**Table 3:** Self-reported prevalence of frequent mental distress (FMD) among older adults living alone versus living in multigenerational families, BRFSS 2016–2020.

	Living Alone		Living in Multigenerational Families		
	% ^1^	[95% CI]	% ^1^	[95% CI]	
65 and older	9.1	[8.8, 9.5]	9.0	[7.6, 10.6]	
Age groups					
65–69	12.8	[11.8, 13.8]	10.5	[8.3, 13.1]	
70–74	10.0	[9.3, 10.8]	12.8	[9.6, 16.8]	
75–79	8.4	[7.6, 9.3]	3.7	[2.2, 6.2]	***
80 and above	7.2	[6.6, 7.8]	4.0	[2.2, 7.0]	**

^1^ Weighted prevalence. CI, confidence interval. ** *p* < 0.05 and *** *p* < 0.01; probabilities compare the FMD among older adults living alone versus living in multigenerational families.

**Table 4:** Logistic regression results—FMD among older adults, BRFSS 2016–2020.

	Model 1				Model 3			
*Unadjusted Models*	OR		[95% CI]		OR		[95% CI]	
Multigenerational (Ref: living alone)	0.98		[0.81,	1.19]				
Multigenerational (Ref: living alone)								
With grandchild only					1.49	*	[0.99,	2.22]
With grandchild, spouse, and/or other adults					0.93		[0.75,	1,15]
	**Model 2**				**Model 4**			
***Adjusted Models***	AOR		[95% CI]		AOR		[95% CI]	
Multigenerational (Ref: living alone)	0.81	*	[0.65,	1.02]				
Multigenerational (Ref: living alone)								
With grandchild only					1.19		[0.75,	1.87]
With grandchild, spouse, and/or other adults					0.77	**	[0.60,	0.99]
Age (5 year age groups)	0.73	***	[0.70,	0.76]	0.73	***	[0.70,	0.76]
Female (Ref: male)	1.04		[0.93,	1.16]	1.03		[0.92,	1.16]
Divorced/widowed/separated/never married	0.88		[0.73,	1.05]	0.86		[0.72,	1.03]
(Ref: married/unmarried couple)								
Race/ethnicity (Ref: NH White)								
NH Black	0.87		[0.72,	1.06]	0.87		[0.71,	1.06]
NH Other	0.92		[0.71,	1.20]	0.92		[0.71,	1.20]
Hispanic	0.90		[0.71,	1.15]	0.91		[0.71,	1.15]
Education (Ref: <high school)								
High school	1.04		[0.88,	1.24]	1.04		[0.88,	1.24]
Attended college or technical school	1.11		[0.93,	1.32]	1.11		[0.93,	1.32]
≥College or technical school	0.99		[0.82,	1.20]	0.99		[0.82,	1.20]
Income (Ref: <$15,000)								
$15,000–24,999	0.93		[0.81,	1.07]	0.93		[0.81,	1.07]
$25,000–34,999	0.80	***	[0.68,	0.94]	0.80	***	[0.68,	0.94]
$35,000–44,999	0.85	*	[0.71,	1.01]	0.85	*	[0.71,	1.01]
≥$50,000	0.72	***	[0.59,	0.87]	0.72	***	[0.59,	0.87]
Chronic conditions	1.34	***	[1.30,	1.39]	1.34	***	[1.30,	1.39]
Physical limitations	1.57	***	[1.51,	1.63]	1.57	***	[1.51,	1.63]
Year: 2020 (Ref: 2016–2019)	1.03		[0.89,	1.21]	1.03		[0.88,	1.20]

OR, odds ratio; AOR, adjusted OR; CI, confidence interval; Ref: reference category. *** *p* < 0.01; ** *p* < 0.05; and * *p* < 0.1.

**Table 5:** Stratified logistic regression results—FMD among older adults living alone (Model 1) versus living in multigenerational families with grandchild, spouse, and/or other adults (Model 2), BRFSS 2016–2020.

	Model 1:				Model 2:			
	Living Alone				Living with Grandchild, Spouse, and/or Other Adults			
	n = 122,246; N = 24,723,912				n = 3046; N = 1,550,179			
	AOR		[95% CI]		AOR		[95% CI]	
Age (5 year age groups)	0.74	***	[0.71,	0.77]	0.56	***	[0.46,	0.70]
Female (Ref: male)	0.99		[0.88,	1.11]	1.97	***	[1.22,	3.19]
Divorced/widowed/separated/never married	0.81	***	[0.67,	0.97]	1.26		[0.77,	2.09]
(Ref: married/unmarried couple)								
Race/ethnicity (Ref: NH White)								
NH Black	0.96		[0.78,	1.18]	0.20	***	[0.09,	0.46]
NH Other	0.97		[0.74,	1.28]	0.25	***	[0.12,	0.52]
Hispanic	0.99		[0.77,	1.27]	0.46		[0.18,	1.17]
Education (Ref: <high school)								
High school	1.05		[0.88,	1.26]	0.90		[0.41,	1.99]
Attended college or technical school	1.11		[0.93,	1.33]	1.04		[0.46,	2.38]
≥College or technical school	0.99		[0.81,	1.20]	1.29		[0.55,	3.05]
Income (Ref: <$15,000)								
$15,000–24,999	0.90		[0.78,	1.04]	2.37		[1.19,	4.74]
$25,000–34,999	0.78	***	[0.67,	0.92]	1.60		[0.64,	4.02]
$35,000–44,999	0.81	***	[0.67,	0.97]	2.30	***	[1.00,	5.30]
≥$50,000	0.71	***	[0.58,	0.87]	1.14		[0.50,	2.61]
Chronic conditions	1.34	***	[1.30,	1.39]	1.44	***	[1.26,	1.65]
Physical limitations	1.58	***	[1.51,	1.64]	1.49	***	[1.26,	1.76]
Year: 2020 (Ref: 2016–2019)	1.01		[0.86,	1.19]	1.50		[0.77,	2.93]

AOR, adjusted OR; CI, confidence interval; Ref, reference category. *** *p* < 0.01

## Data Availability

Data are available from the authors upon request.
